# The development of the EUropean Physical Activity Determinants framework for Adolescents (EU-PAD-A): a mixed-methods concept mapping study within the DE-PASS COST action

**DOI:** 10.1186/s12966-026-01878-0

**Published:** 2026-02-05

**Authors:** Andrea Fusco, Cristina Cortis, Paul Jarle Mork, Daniele Conte, Francesca Di Rocco, Emanuel Festino, Pascal Izzicupo, Stefania Orrù, Anne Lovise Nordstoga, Olga Papale, Marianna De Maio, Valentina Presta, Padraic Rocliffe, Laura Capranica, Ciaran MacDonncha, Giancarlo Condello

**Affiliations:** 1 Department of Medicine and Aging Sciences, University “G. d’Annunzio” of Chieti-Pescara, Chieti, Italy; 2 Department of Human Sciences, Society and Health, University of Cassino and Lazio Meridionale, Cassino, Italy; 3 Department of Public Health and Nursing, Norwegian University of Science and Technology, Trondheim, Norway; 4 Department of Movement, Human and Health Sciences, University of Rome “Foro Italico”, Rome, Italy; 5 Department of Coaching Science, Lithuanian Sports University, Kaunas, Lithuania; 6 Department of Medical, Human Movement and Well-Being Sciences, University of Naples Parthenope, Naples, Italy; 7 CEINGE Biotecnologie Avanzate “F. Salvatore”, Naples, Italy; 8 Department of Neuromedicine and Movement Science, Norwegian University of Science and Technology, Trondheim, Norway; 9 Department of Medicine and Surgery, University of Parma, Parma, Italy; 10 Department of Physical Education and Sport Sciences, Physical Activity for Health Cluster, Health Research Institute, University of Limerick, Limerick, Ireland

**Keywords:** Adolescent behaviour, Concept mapping, Health promotion, Social environment

## Abstract

**Background:**

Most European adolescents do not meet recommended levels of physical activity (PA), increasing their risk of long-term health issues and contributing to the burden of non-communicable diseases. To address this public health challenge, there is a need for evidence-based, age-specific frameworks that identify the key determinants influencing PA behaviours during adolescence, a critical developmental stage for habit formation.

**Methods:**

This study developed the EUropean Physical Activity Determinants framework for Adolescents (EU-PAD-A) and identified the most important and modifiable factors that can inform targeted interventions and public policy. Using Group Concept Mapping analysis, 240 experts contributed to the identification, sorting, and rating of PA determinants for adolescents.

**Results:**

A total number of 110 determinants were grouped into nine thematic clusters. Determinants were rated for importance and modifiability, with 53% (*n* = 58) scoring high on both dimensions. To refine priorities for policy and practice, a focus group of experts and a survey involving 60 policy makers and stakeholders were conducted. This process led to the identification of the top 10 actionable determinants, defined as those rated highly for importance and modifiability by experts and for impact and feasibility by policy makers and stakeholders, including availability of indoor and outdoor PA facilities, beliefs about PA, and inclusive school programs.

**Conclusions:**

The results highlight school, family, and socio-cultural environments as critical settings for intervention. The EU-PAD-A framework offers a novel, interdisciplinary, and age-specific tool for understanding and addressing the complex drivers of adolescent PA. It provides concrete, consensus-based guidance for designing policies and programs that are feasible, impactful, and grounded in scientific evidence. As such, it represents an important step toward improving PA levels and reducing sedentary behaviour among adolescents across Europe.

**Supplementary Information:**

The online version contains supplementary material available at 10.1186/s12966-026-01878-0.

## Introduction

Most children and adolescents do not meet recommendations for physical activity (PA) [1], sleep [2], and diet [3, 4]. These unhealthy behaviours often transfer into adulthood and older age, with only about 6% of the general adult population in Europe meeting recommendations for PA, fruit and vegetables intake, alcohol consumption, and smoking [1, 5–7]. 

The rise in inactivity contributes to higher healthcare costs and increased mortality from non-communicable diseases [8–10]. However, this trend could be mitigated with adequate and sustained public investments in interventions to increase PA levels [11, 12]. Such interventions should particularly target adolescents, as their adoption of healthy behaviours is associated with medium- and long-term health outcomes [13]. Moreover, to be effective it is necessary that the development of such interventions incorporate knowledge about factors that may have direct causal effects on PA level (i.e., determinants) versus factors with other types of association with PA level (i.e., correlates, mediators, and moderators) [14].

The first European Joint Programming Initiative Healthy Diet for a Healthy Life (JPI HDHL) “DEterminants of DIet and Physical Activity (DEDIPAC)” project aimed to identify key determinants of an active lifestyle [15]. Systematic reviews within the DEDIPAC project found limited evidence on the influence of possible determinants on PA level across the life course, highlighting the need for further research targeting specific settings [16–22]. Furthermore, the DEDIPAC project engaged PA experts to develop the EUropean framework of PA Determinants (EU-PAD framework) using the concept mapping methodology [23]. This framework included six clusters within the personal (intra-personal context and well-being; family and socio-economic status) and societal areas (policy and provision; cultural context and media; social support and modelling; supportive environment). The rating task identified 25 factors as the most modifiable and influential across the life course, with 16 considered to be of high research priority. However, the EU-PAD framework covered all life stages and was not tailored to specific settings.

In 2022, the European Cooperation in Science and Technology (COST) funded the 3-year international and transdisciplinary action “DEterminants of Physical ActivitieS in Settings (DE-PASS, CA19101; https://depass.eu/) to be an effective knowledge transfer platform. The DE-PASS COST action established a network of 319 researchers and experts from 37 countries who jointly worked to synthesize high-quality interdisciplinary research in relation to different age groups. The aim was to identify, measure, and understand the determinants that promote, maintain or inhibit PA in different settings across the lifespan. Moreover, DE-PASS aimed to translate the best evidence statements (BESt) related to promoting PA to help policymakers achieve greater health impact. While some determinants, such as gender and age, are non-modifiable, others, like family support and motivation, can be influenced by targeted interventions. A systematic literature review and meta-analysis focused on determinants of adolescents PA and sedentary behaviour in various settings. The findings from randomized controlled trials indicated that parental influence and social support have varying degrees of impact on reducing sedentary behaviour and increasing PA [24]. However, there is a need for further synthetisation of modifiable determinants of PA in adolescents that can be implemented in targeted interventions.

The twofold aim of this study was therefore to (1) develop a EUropean Physical Activity Determinants framework for Adolescents (EU-PAD-A), and (2) identify the most influential and modifiable determinants to inform effective public policies and interventions. By focusing on adolescents, the study seeks to provide policy makers with actionable insights for designing interventions that promote sustainable healthy lifestyles.

## Methods

### Group concept mapping

The Group Concept Mapping (GCM) approach was applied to reach the aim of the study. Concept Systems^®^ GroupWisdom™ software (Concept Systems, Incorporated, NY, USA) was used for the entire process. GCM is a mixed-method approach that supports the creation of valid and reliable metrics and scales from a diverse group of participants with various disciplinary backgrounds. GCM methodology collects, integrates, and represents both visually and numerically the collective thoughts of a group of relevant and expert stakeholders concerning a complex social phenomenon. The methodology includes the preparation, generation, structuring, analysis, and interpretation phases [25–27]. A detailed description of each phase of the GCM methodology is provided in Table 1, specifying the activities and identifying the participants engaged at each stage.

**Table 1:** Description of activities and participants for each phase of the group concept mapping (GCM) methodology

GCM phase	Activity	Participants
Preparation	Develop recruitment protocol Finalize Focus Prompt	Project Leaders (C.M. and L.C.) Work Package Leaders (C.C. and P.J.M.) Deliverable Leaders (A.F. and G.C.) Research Team Members (A.L.N. and P.R.)
Generation	Literature review Brainstorming phase Generate a final list of determinants	Deliverable Leaders (A.F. and G.C.) Research Team Members (A.L.N. and P.R.) Experts
Structure	Sorting of determinants Rating for modifiability and importance	Experts
Analysis	Multi-dimensional scaling Cluster analysis Review point maps and proposed clusters Identify ideal cluster map and name clusters	Project Leaders (C.M. and L.C.) Work Package Leaders (C.C. and P.J.M.) Deliverable Leaders (A.F. and G.C.)
Interpretation	Draft a Concept Map based on the selected clustering solution	Work Package Leaders (C.C. and P.J.M.) Deliverable Leaders (A.F. and G.C.)

The Institutional Review Board of the Department of Human Sciences, Society and Health of the University of Cassino and Lazio Meridionale approved the research ethics and associated protocols for this study (Approval No.: 9407; dated 8 March 2023).

#### Participants

A total of 240 experts contributed to at least one phase of the GCM (*n* = 38 in brainstorming; *n* = 95 in sorting; *n* = 107 in rating; some overlap across phases is expected). Sample sizes for the last 2 phases highly exceed the average number of responders reported in a study assessing the quality and rigor of the concept mapping methodology [28]. Participants have been recruited by a convenience sampling within the DE-PASS network and external institutional network. All of them were involved in PA- and health-related work as researchers and/or policymakers with a minimum of 5 years of experience. A greater proportion of participants from Academic and Research Positions (68%) was recruited for the brainstorming phase, whilst their proportion was lower for the sorting (33%) and rating (36%) phase. This imbalance was required to ensure an adequate generation of determinants with the higher contribution of academic members and researchers from the field of PA and health. In total, 34 nations across the European Union (EU) and outside the EU were involved, with a prevalence of EU nationalities (an average of 81% across the three GCM phases). The discrepancy between EU and non-EU countries reflects the nature of the COST action, which takes place in Europe and established a network mainly among European countries, the objective of the study, which was the development of a European framework, and the lower response rate of non-EU participants. However, the final sample constituted a mix of experts from both national and international backgrounds and with a comprehensive multi-disciplinary expertise and stakeholder groups. Therefore, Academic Professors and Researchers, Medical Doctors, Physical Education Teachers, Deputy Mayors, Climatologists, Environmental Engineers, Cardiologists, Lawyers, Local Administrators, Sport Managers, Marketing Freelancers, Graduate and Undergraduate Students, Municipality Counselors, PhD Students, Project Managers, Social Media Managers, Specialists at the Ministry of Education, Sport Federation Directors, Travel Agents, Technical Officers and Sport Coaches were engaged.

All respondents remained anonymous, and the following demographics were gathered from the participants: age, gender, parent (whether the participant has children or the participant’s status as a guardian), job title and field, and nationality (Table 2).

**Table 2:** Demographic characteristics of participants for each phase

Characteristic	Brainstorming (*n* = 38)	Sorting (*n* = 95)	Rating (*n* = 107)
Age (years)	45 ± 9	38 ± 13	39 ± 13
Gender	Male 50%, Female 50%	Male 59%, Female 41%	Male 55%, Female 45%
Parent status	Yes 58%, No 42%	Yes 39%, No 61%	Yes 38%, No 62%
Nationality	EU 74%, Non-EU 26%	EU 86%, Non-EU 14%	EU 83%, Non-EU 17%
Current role (%)			
Academic/Research	68%	33%	36%
Medicine	21%	5%	4%
Physical Education	5%	5%	7%
Governance/Administration	5%	9%	9%
Law	–	5%	5%
Specialized/Other	–	5%	5%
University students	–	37%	35%
Job field/sector (%)			
Healthcare	29%	25%	28%
Sport & Exercise	39%	46%	46%
Education	13%	7%	8%
Rehabilitation	8%	–	–
Governance/Industry	–	16%	14%
Environmental/Urban	10%	5%	4%

#### Preparation

During the preparation phase (January 2023), the finalization of the focus prompt was executed to facilitate the recruitment of the participants and idea generation in the subsequent brainstorming phase. During the brainstorming phase, the following focus prompt was provided: “In the context in which female and male adolescents (13–19 years) live, play and are physically active such as the home, school and the wider community, a specific factor that may, in a positive or negative way, influence their physical activity behaviour, would be…”.

#### Generation

To gather a comprehensive list of determinants, two methods were employed: (a) analysis of the findings from the umbrella systematic reviews conducted within the DEDIPAC project [16–22]; (b) ideas generated and synthesized from brainstorming sessions involving PA experts (see Tables 1 and 2 for details), followed by a synthesis of the generated ideas in a list of relevant determinants, which may influence female and male adolescents’ PA behaviours in the context in which they live, play and are physically active, such as the home, school and the wider community.

During the generation phase, an online brainstorming session was distributed to different participants within the European Union (see Tables 1 and 2 for details), who were recruited via institutional and professional associations from March to May 2023. To analyse the open-ended responses of the brainstorming phase, three researchers (A.L.N.; P.R.; A.F.) initiated the process by creating an initial codebook based on the Focus Prompt answers. Subsequently, two researchers (A.L.N.; P.R.) independently coded each participant’s response, refining the codebook as necessary. This review aimed to ensure clarity, avoid repetitions, and decide if any of the identified determinants needed to be broken down further. Expert researchers with a deep knowledge in the subject area and a good command of English were invited to evaluate and provide insights on the determinants. A 5-point Likert scale was used to evaluate the clarity (ease of understanding) for each determinant, ranging from 1 (unclear) to 5 (clear). Researchers were provided the opportunity to comment on each determinant, offering insights regarding its relevancy, representativeness, rateability, and the saturation of the topic. This feedback was intended to refine and enhance the quality of the factor analysis. In accordance with established guidelines, determinants that received an evaluation score below 3 were either revised to improve clarity or entirely removed from the list [29]. Discrepancies were discussed collaboratively until a consensus was achieved with the supervision of the third researcher (A.F.).

After completing the processes, a comprehensive and exhaustive list of 110 determinants affecting adolescents’ PA behaviours, as identified by different categories and stakeholders, was uploaded to Concept Systems software for the subsequent structuring phase (sorting and ratings).

#### Structure

The structure phase comprised two distinct activities: sorting and rating. For this phase, a new and larger cohort of experts, was recruited from June 2023 to December 2023. After collecting participant information and obtaining their consent, participants were given access to the final list of determinants through the Concept Systems software. Initially, the determinants were presented in a random order, and participants used the Concept Systems software to categorize the determinants identified in the generation phase by sorting them based on perceived similarities. Each determinant was labelled with a unique code to indicate its origin, whether from the brainstorming phase or the literature review. Participants were also provided with an identification code to guarantee a consistent uniform interpretation. Participants could create any number of categories, with at least two categories required. Each category had to be named to reflect its theme or content. Each determinant was assigned to only one category, ensuring that no determinant belonged to multiple categories. If a determinant appeared distinct or unique, it could stand alone within its own category. Participants had the flexibility to name the categories either immediately or during the overall sorting process. After completing the initial organization, participants could review and adjust the categories as needed. Incomplete sorting tasks were excluded from the final analysis. All sorting submissions were manually reviewed by an experienced researcher with formal training and licensure in concept mapping (A.F.) to verify adherence to the sorting task guidelines. Only fully completed and compliant sorting datasets were retained for analysis.

The rating phase was conducted from January 2024 to June 2024 via an online survey, allowing participants to evaluate the importance and modifiability of the determinants on a scale of 1 to 6 (1 indicating not important/not modifiable and 6 indicating very important/very modifiable). Reminder emails were sent 3 times to non-responding participants to minimize non-response bias and ensure a higher response rate. Incomplete rating questionnaires were manually reviewed by the same experienced researcher (A.F.) to verify completeness and compliance with the rating task instructions. Only completed rating questionnaires were retained for analysis, and determinant-level mean ratings were calculated using all available responses, as accommodated by the Concept Systems software.

#### Concept mapping analysis

The analysis used a mixed-methods approach, combining qualitative data gathered from the literature reviews and expert stakeholders with quantitative data from participants sorting and ratings. Descriptive statistics, including means and standard deviations, were computed for the rating of the determinants’ importance and modifiability to provide a quantitative overview of the data. The responses were visually represented on a two-dimensional concept map using multi-dimensional scaling and hierarchical cluster analysis through Concept Systems software. The multi-dimensional scaling analysis is a statistical analysis commonly used to visualize similarities or dissimilarities between data points. Starting from proximity data as input, it allows determining the position of each point in a two-dimensional space, that is translated in a concept map, where the distances between the points in the new space closely reflect the original data. The generated concept map facilitates the visual interpretation of relationships and clustering among the data points of each participant contributing to the study. This analysis helped organizing the sorting data spatially, showing patterns and connections. Determinants that were positioned closer together on the concept map were sorted together more frequently, indicating a higher degree of similarity, whereas those that were further apart were sorted together less frequently, suggesting less association between them. Internal validity was assessed through a stress value, which indicates how well the multidimensional scaling configuration represented the data relationships. A previous study indicated that an average stress values equal to 0.28 (range from 0.17 to 0.34) is typical for concept mapping analyses [28]. This procedure was further improved by determining each determinant’s bridging value, a measure ranging from 0 to 1 that indicates the strength of a determinant’s association with its near determinants. Bridging values were calculated for each determinant as a normalized index ranging from 0 to 1, derived from the aggregated sorting (co-occurrence) data and the resulting multidimensional scaling configuration. This index reflects the extent to which a determinant was consistently sorted with items within its own cluster (low bridging values, indicating anchor determinants) or, conversely, with items across multiple clusters (high bridging values, indicating bridging determinants). For example, lower bridging values denote anchors, indicating determinants with strong connections to their immediate location on the map, while higher values identify bridges, representing determinants with more spread associations across different areas of the cluster map. These relationships were visually examined through a spanning analysis, which facilitated conceptual interpretation by illustrating weighted connections between items.

The concept map also aims to explain the conceptual framework by segmenting the determinants in 2D representation and clustering them based on their proximity. The determinants were grouped into clusters using hierarchical cluster analysis, which organized data based on the similarities among the determinants, facilitating clear categorization. Each cluster represented conceptual relationships among the determinants, highlighting how closely similar the grouped determinants were in terms of their content and context. Clusters of determinants positioned further apart demonstrate more independence compared to those nearer to each other. Three researchers (A.F.; G.C.; C.C.) collaborated to evaluate various candidate concept maps, ranging from 6 to 14 clusters for each cluster solution, to identify the configuration most suitable for the study rationale. It was previously indicated that a range from 6 to 14 clusters for a map is common with a median value of 9 [28]. Subsequently, a group of expert researchers (A.F.; G.C.; C.C.; P.J.M.; C.M.; L.C.) reviewed each cluster to determine the final number of clusters, ensuring that each represented a distinct meaning. This process was guided by the objective of clearly defining and differentiating the determinants that may influence PA behaviours in adolescents. Furthermore, the same group of researchers assigned a descriptive label to each cluster, based on the participants’ proposed labels. This cluster labelling process was conducted in July and August 2024. A final interpretation phase, involving the same group of expert researchers reviewed the draft concept maps and associated ratings data. Various concept map visualizations were discussed to consider the suitability of each cluster name and examine the determinant ratings. This stage was conducted in September and October 2024.

Determinant ratings from the participants were further analysed to enhance the concept mapping interpretation using visualization techniques, namely bivariate graphs divided into quadrants by mean values (Go-Zones). Importance ratings were plotted on the x-axis and modifiability ratings on the y-axis. The Go-Zones map was divided into four quadrants by setting the vertical cut-point at the mean importance value and the horizontal cut-point at the mean modifiability value calculated across all determinants. The minimum, maximum, and mean values for both importance and modifiability ratings were used to describe the distribution of determinants within the four quadrants. Each determinant was plotted as a data point on the scatter plot to visually represent its relative position in terms of importance and modifiability. This visualization facilitates the identification of determinants that are simultaneously rated as highly important and highly modifiable, which may represent priority targets for policy and intervention efforts. Moreover, the Pearson correlation coefficient (r) was calculated to quantify the relationship between importance and modifiability ratings across determinants.

### Influential determinants for policy makers and stakeholders

The secondary objective of this study was reached by selecting the most relevant determinants for policy makers and stakeholders from the 110 determinants developed during the GCM. Specifically, a focus group technique encompassing nine experts in PA was used for the selection of the most relevant determinants and it was conducted in November and December 2024 by online meetings. The nine experts were selected among the authors: Project Leaders C.M. and L.C.; Work Package Leaders C.C. and P.J.M.; Deliverable Leaders A.F. and G.C.; Research Team Members D.C., A.L.N. and O.P. In the first stage of the focus group activity, each expert was required to personally identify from the original list of 110 determinants the most influential and modifiable determinants to inform effective public policies and interventions. Subsequently, an in-depth discussion occurred in an online focus group setting led by an experienced facilitator. This combination allowed us to reduce the potential number of determinant factors which could contribute to effective public policies and intervention design. During the discussion, firstly determinants indicating a similar meaning were considered (i.e., “Availability of indoor and outdoor physical activity facilities” and “Indoor and outdoor sports facilities availability”) and only one of those was selected based on the decision of the focus group. Moreover, during the discussion, determinants deemed too generic and/or not relevant for policy makers and stakeholders (e.g., stress, genetic aspects) were excluded based on unanimous decision of the experts. The final list consisted of 36 determinants.

The following step encompassed the adoption of a two-step cluster analysis for these 36 determinants using determinants’ importance and modifiability ratings generated during the GCM. This statistical approach was used to group the determinants in two clusters characterized by high and low importance and modifiability. Furthermore, the two-step cluster analysis identified also the predictor importance, showing that determinants’ importance (value = 1) was the predictor better differentiating between clusters compared to modifiability (value = 0.8). Predictor importance has been assessed by measuring how much each variable improves cluster separation in a likelihood-based model, then scaling those contributions relative to the strongest predictor. The analysis provided two clusters based on Schwarz’s Bayesian Criterion: the high importance (mean ± SD = 4.15 ± 0.26) and modifiability (mean ± SD = 3.85 ± 0.53) cluster comprised 29 PA determinants, while the low importance (mean ± SD = 3.31 ± 0.45) and modifiability (mean ± SD = 2.72 ± 0.43) cluster included 7 PA determinants. From the high importance and modifiability cluster, the 10 PA determinants with the highest values of importance and modifiability were retained for the development of the questionnaire to be administered to a sample of policy makers and stakeholders. The 10 selected determinants were: “Availability of indoor and outdoor physical activity facilities”, “Belief about physical activity”, “Inclusive programs in school”, “Social inclusion”, “Perceived barriers to physical activity”, “Provision of school facilities resources”, “Incentives to be physically active”, “Maintenance of school facilities and equipment”, “Social economic status”, and “Provision of sports clubs in schools”.

An online questionnaire aimed at assessing the potential impact and feasibility to implement the 10 determinants was created for a specific sample of policy makers and stakeholders (*n* = 60). The online survey was completed by 60 policy makers and stakeholders who were contacted via email and social media and voluntarily agreed to participate. Participants were recruited through the DE-PASS network and through the personal professional networks of DE-PASS members. Policymakers and stakeholders were selected based on their expertise and professional roles, including academics, sport managers, politicians involved in sport-related issues at national, regional, and international levels, representatives of non-governmental organizations, and school leaders. To ensure that all respondents identified themselves as policymakers and/or relevant stakeholders for the project, participants were required to answer the following screening question prior to completing the online questionnaire: “Are you currently, or have you previously been, working or acting as a policymaker and/or stakeholder?”. The following prompts developed by the 9 experts during the focus group phase, guided responses for all 10 determinants; in addition, the questionnaire established the role of the participant regarding physical activity policy and provision:

1. “In the context in which female and male adolescents (13–19 years) live, play and are physically active such as the home, school and the wider community, a specific factor that has the potential to positively impact on individual and/or population physical activity behaviour, would be…”.
2. “In the context in which female and male adolescents (13–19 years) live, play and are physically active such as the home, school and the wider community, a specific factor that is feasible to implement via policy and practice to positively influence their physical activity behaviour, would be…”.

Each determinant was rated on a 6-point Likert scale for the first prompt (1 no potential impact, 6 great potential impact) and the second prompt (1 not feasible, 6 very feasible). The questionnaire was designed in a user-friendly manner with online survey, with completion only requiring about 5 min.

The two-step cluster analysis was performed using the SPSS software (version 29.0.1, IBM). Descriptive statistics including the frequency of occurrence were used to express the percentage of responses for each determinant for the impact and feasibility scales.

## Results

**Fig. 1:**
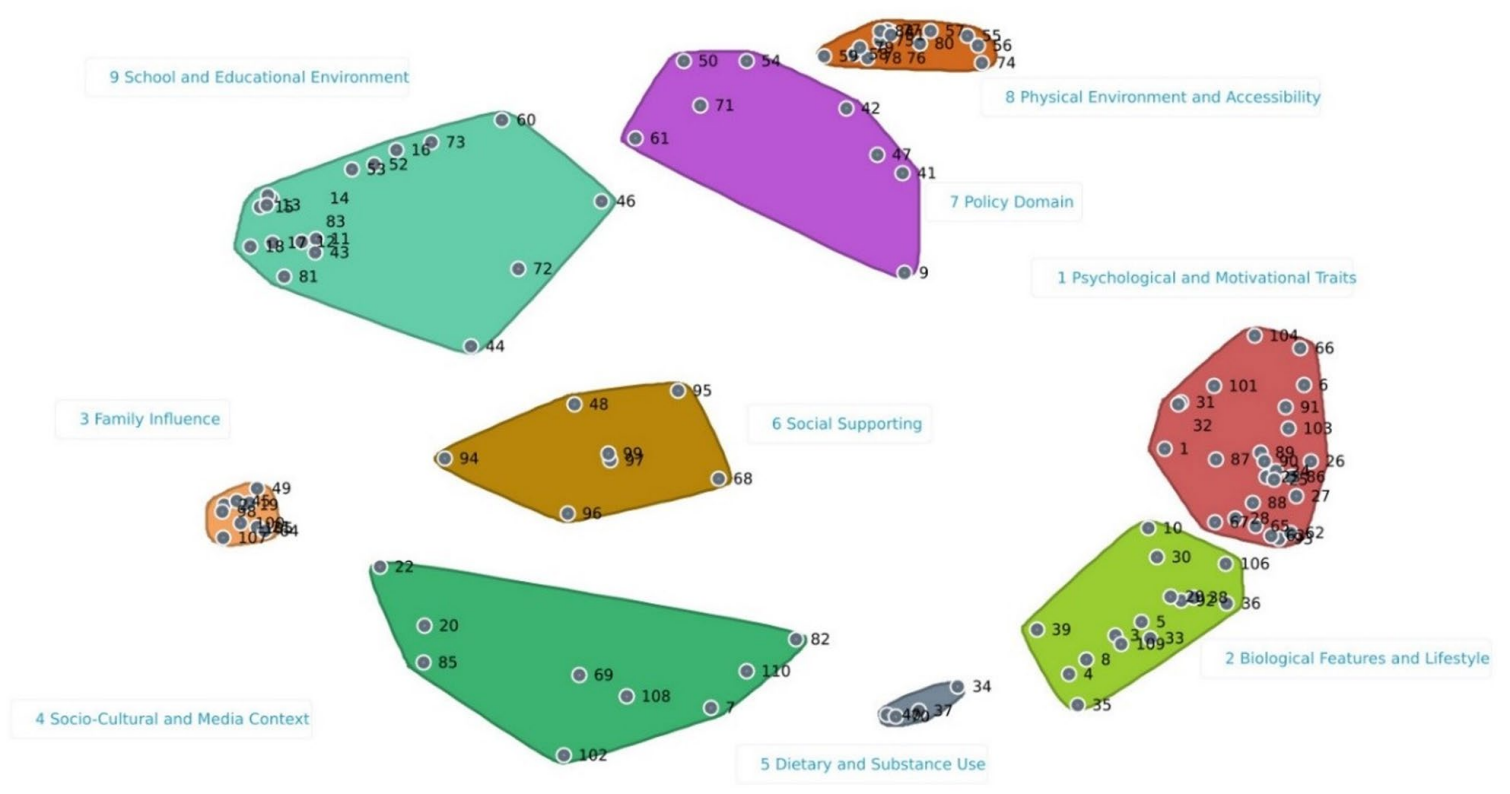
Concept map that organizes 110 determinants into 9 clusters

The two-dimensional map revealed a stress value of 0.26, which is considered satisfactory based on Rosas and Kane [28], indicating the map accurately represents the participants opinion and it is not randomly generated. The EU-PAD-A framework displays the 110 determinants into 9 clusters (Fig. 1). The map solution with 9 clusters was selected because of a better stress value and aggregation of determinants and to avoid an excessive fragmentation of clusters. The cluster names were discussed and agreed by 5 expert researchers, and the following labels were chosen: Cluster 1 “Psychological and Motivational Traits”; Cluster 2 “Biological Features and Lifestyle”; Cluster 3 “Family Influence”; Cluster 4 “Socio-Cultural and Media Context”; Cluster 5 “Dietary and Substance Use”; Cluster 6 “Social Support”; Cluster 7 “Policy Domain”; Cluster 8 “Physical Environment and Accessibility”; Cluster 9 “School and Educational Environment”.

Spanning analysis was conducted systematically for all determinants to identify cross-cluster connections based on aggregated sorting data. Cluster 4 “Socio-Cultural and Media Context” had the highest bridging value (average 0.67), with “Usage of fitness tracker devices” showing the highest bridging value (average 1.00) and “Exposure to social media” demonstrating the lowest bridging value (average 0.66). To facilitate interpretation, an illustrative example is presented for the determinant “Usage of fitness tracker devices”, which showed numerous strong connections both within its primary cluster and with determinants belonging to other clusters, particularly Clusters 1, 2, 5, 6, 7, and 8. For instance, this determinant was frequently sorted together with “Previous experiences in sport” (Cluster 1: Psychological and Motivational Traits; 33 co-occurrences) and “Use of digital devices” (Cluster 4: Socio-Cultural and Media Context; 56 co-occurrences), highlighting its cross-cutting role across multiple thematic domains (Additional Fig. 1). The spanning analysis revealed the bridging value for the clusters and for each determinant within clusters.

Clusters 8 “Physical Environment and Accessibility” displayed the lowest bridging value (average 0.1). The spanning analysis map revealed that the determinants within this cluster were more frequently grouped together, indicating that they are more anchors than other determinants (Additional Fig. 2). Determinants within Cluster 8 exhibited bridging values ranging from the lowest (“Walkability and safety of sidewalks/trails”) to the highest (“Active means of transportation use”). For example, the determinant “Walkability and safety of sidewalks/trails” demonstrated strong associations with other determinants of the same cluster, such as “Streets characteristics”, “Urban design and land use”, “Proximity of parks”, and “Environmental barriers to active travel”.

**Fig. 2:**
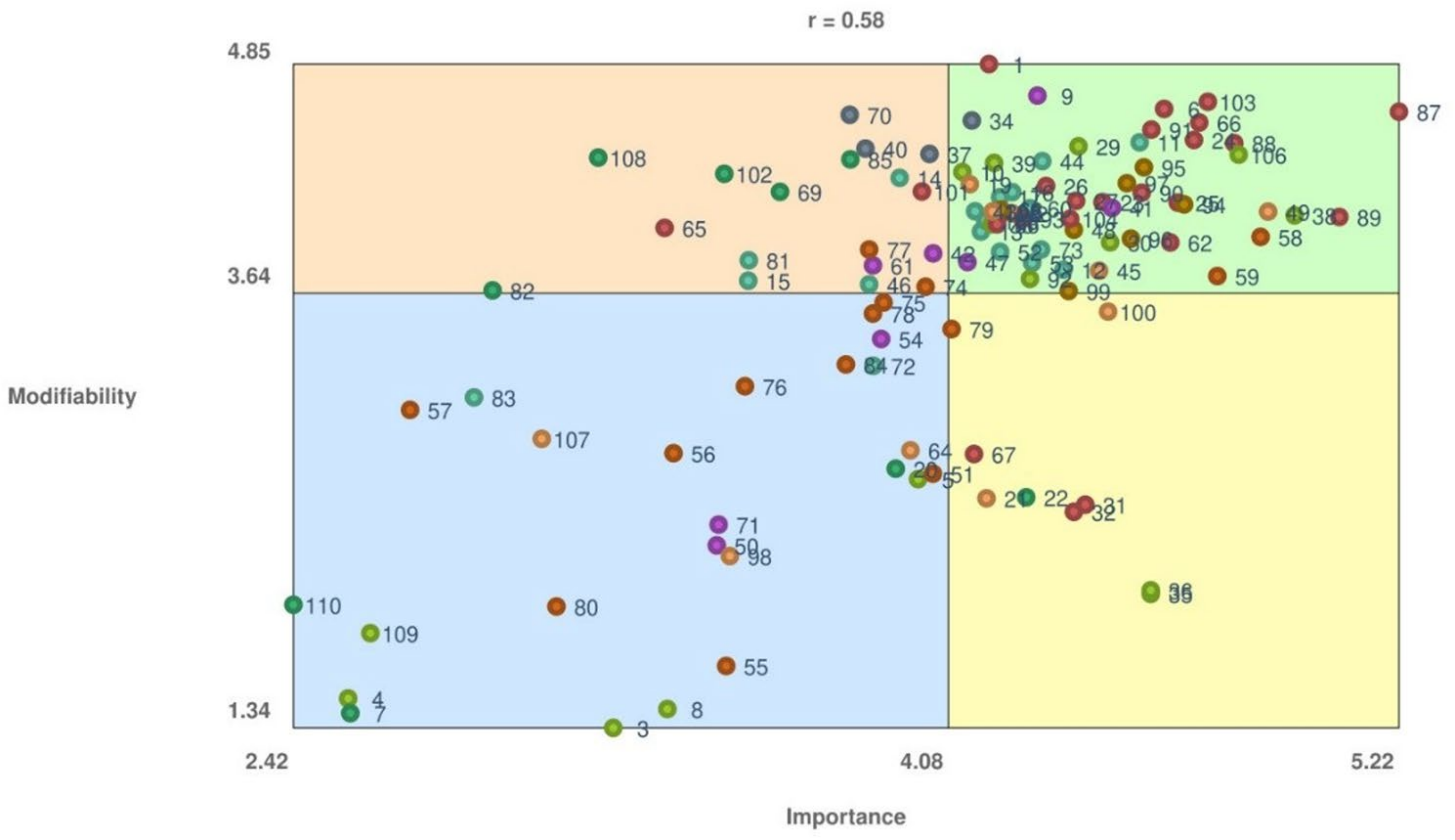
Go-Zones map of importance and modifiability ratings for the 110 determinants

The Go-Zones analysis revealed an *r* = 0.58, indicating that the determinants perceived highly important tend also to be considered highly modifiable (Fig. 2). This alignment may reflect consistent expert beliefs about policy and practice leverage. The quadrants were defined using the overall mean values across all determinants as cut-points: importance = 4.08 (range 2.42–5.22) and modifiability = 3.64 (range 1.43–4.85). Accordingly, “high” importance refers to determinants scoring above 4.08 on the 1–6 scale, while “high” modifiability refers to determinants scoring above 3.64. Determinants in Quadrant IV (*n* = 58) therefore represent items simultaneously rated above the mean on both dimensions. “Enjoyment” exhibited the highest importance (average 5.22), whereas “Knowledge/awareness of health benefits” the highest modifiability (average 4.85). In contrast, “Religion” had the lowest importance value (average 2.42), whereas “Age” was considered the least modifiable (average 1.34). Clusters mostly represented are Cluster 6 “Social Support” (100%), Cluster 1 “Psychological and Motivational Traits” (80%), Cluster 9 “School and Educational Environment” (67%), Cluster 2 “Biological Features and Lifestyle” (53%), and Cluster 3 “Family Influence” (50%). None of the determinants in Cluster 4 “Socio-Cultural and media Context” is included in Quadrant IV.

The responses of 60 policy makers and stakeholders for the potential impact and feasibility of the 10 determinants are shown in Table 3. “Availability of indoor and outdoor physical activity facilities” and “Belief about physical activity” were the two determinants with the greatest potential impact (61.7% and 40%, respectively). Furthermore, “Availability of indoor and outdoor physical activity facilities” and “Inclusive programs in schools” were rated as the determinants with the highest degree of feasibility (41.7% and 38.3%, respectively). Spanning analysis revealed that “Availability of indoor and outdoor physical activity facilities”, “Belief about physical activity”, and “Inclusive programs in schools” are anchors, with bridge values equal to 0.10, 0.28, and 0.17, respectively (Additional Figs. 3, 4 and 5).

**Table 3:** Percentage of responses for the potential impact and feasibility of the 10 selected determinants

	Score	Availability of indoor and outdoor physical activity facilities	Belief about physical activity	Inclusive programs in school	Social inclusion	Perceived barriers to physical activity	Provision of school facilities resources	Incentives to be physically active	Maintenance of school facilities and equipment	Social economic status	Provision of sports clubs in schools
Impact	6	61.7%	40.0%	35.0%	30.0%	16.7%	36.7%	23.3%	33.3%	18.3%	33.3%
	5	26.7%	31.7%	33.3%	26.7%	31.7%	36.7%	38.3%	28.3%	30.0%	25.0%
	4	1.7%	16.7%	16.7%	30.0%	33.3%	11.7%	23.3%	23.3%	25.0%	21.7%
	3	8.3%	10.0%	11.7%	10.0%	10.0%	15.0%	8.3%	10.0%	8.3%	11.7%
	2	1.7%	1.7%	3.3%	3.3%	5.0%	0.0%	6.7%	5.0%	15.0%	5.0%
	1	0.0%	0.0%	0.0%	0.0%	3.3%	0.0%	0.0%	0.0%	3.3%	3.3%
Feasibility	6	41.7%	15.0%	38.3%	21.7%	11.7%	33.3%	21.7%	30.0%	11.7%	21.7%
	5	26.7%	48.3%	25.0%	28.3%	33.3%	36.7%	28.3%	26.7%	23.3%	30.0%
	4	6.7%	11.7%	23.3%	23.3%	31.7%	11.7%	23.3%	26.7%	15.0%	23.3%
	3	18.3%	15.0%	10.0%	20.0%	13.3%	11.7%	16.7%	13.3%	18.3%	13.3%
	2	6.7%	8.3%	3.3%	6.7%	8.3%	6.7%	10.0%	3.3%	18.3%	10.0%
	1	0.0%	1.7%	0.0%	0.0%	1.7%	0.0%	0.0%	0.0%	13.3%	1.7%

6 = great potential impact/feasibility; 1 = no potential impact/feasibility

The complete determinants list is provided as additional material with the average value for importance, modifiability, bridging, and Go-Zones quadrant position (Additional Table 1).

## Discussion

Scientific evidence should guide policy makers on the most effective determinants of health-related behaviours, especially in relation to the diverse settings (i.e., family, school, social networks) where PA habits are adopted [30]. In contrast, dedicated frameworks specific for age groups would provide a foundation for understanding the interactions among PA determinants and direct policy makers towards sustainable interventions with long-term benefits. To reach this purpose, the EU-PAD-A framework was specifically tailored for adolescents and was built considering potential settings where adolescent PA behaviours could be adopted. Hence, the initiatives of policy makers and stakeholders should primarily be addressed within those settings. Nonetheless, it should be acknowledged that the adolescents did not participate in any phase of the GCM, since the framework has been built only with the contribution of adult stakeholder inputs. This choice was based on the need to generate, rate, and sort the determinants from adults with the required- knowledge, experience, and competence rather than the lived experience of adolescents.

The framework allows the identification of different settings and areas. Specifically, three main settings appear evident: School, within Cluster 9 “School and Educational Environment”; Family, within Cluster 3 “Family Influence”; Socio-cultural environment, within Cluster 4 “Socio-Cultural and Media Context” and Cluster 6 “Social Support”. The remaining clusters can be grouped into two areas: Cluster 1, 2 and 5 could represent the individual area encompassing psychological, biological, and behavioural traits; Cluster 7 and 8 represent the political and physical environment area. Those areas are commonly represented when PA and sedentary behaviours are examined [23, 31–33].

The importance of the emerging settings is also emphasized by several determinants rated with high importance and modifiability values and included in quadrant IV of the Go-Zones. Unless for Cluster 4, at least 50% of the determinants in Cluster 3, 6, and 9 are included in quadrant IV. These determinants serve as actionable elements for policy interventions.

A recent DE-PASS meta-analysis on PA behaviours in adolescents summarized evidence related to school settings [24]. However, school-based PA interventions have shown limited impact on behavioural changes in adolescents [31, 34], even if an improvement in PA during physical education classes could be achieved [35]. Actually, physical education within school curricula remains a critical issue, with a different number of hours in each country [36] and with a risk of marginalization caused by school management, teachers’ responsibility, and parents’ misconceptions [37]. In the current analysis, “Inclusive programs in school” received the highest ratings for both importance (average 4.56) and modifiability (average 4.43). This underscores the need for structured and innovative actions to create PA programs that are available and accessible to all students. The role of schools in promoting healthy behaviours among adolescents was further emphasised by the selection of four school-related determinants (“Inclusive programs in school”, “Provision of school facilities resources”, “Maintenance of school facilities and equipment”, “Provision of sports clubs in schools”) among the top 10 determinants identified by experts and evaluated by policy makers and stakeholders. Notably, over 75% of the respondents rated “Inclusive programs in school” as highly feasible (score ≥ 4). A comprehensive approach to PA development in schools has previously been put forward as high priority [38] and further discussed by the International Society of Physical Activity and Health [39], calling for a multidimensional involvement of society to reduce sedentary behaviour and increase PA behaviours. Initiatives should include regular physical education within curricula, suitable physical and social environments, actions before, during, and after school hours, and the involvement of staff, families and the wider community [24].

The framework identified family as an important setting, with “Parental support” being the determinant with the highest ratings for both importance (average 4.89) and modifiability (average 4.07). Generally, the role of parents in supporting their children’s participation in PA is widely debated in isolation or included in broader determinants categories, as “Social support” [40], “Supportive behaviour from significant” [20], “Family support” [24], though evidence in adolescents remains limited. The potential role of parents in influencing adolescents’ participation in PA is also related with “Belief about PA” (Cluster 1 of the current framework), which demonstrated high importance (average 4.59) and modifiability (average 4.50). “Belief about PA” is also one of the 10 determinants selected for policy makers and stakeholders, receiving high responses for its impact (88% of total responders rated it with a score ≥ 4). Although no determinants from Cluster 3 were selected and evaluated by policy makers and stakeholders, “Parental support” and other family-related determinants remain crucial determinants for adopting healthy behaviours during the early stages of life, which could be sustained, cultivated, and transferred throughout the life course. Parents’ values, beliefs, and perceptions of PA and sport participation are fundamental for shaping their children’s future lifestyle [41]. Recognizing that parents may not always fully understand their role in inspiring and sustaining their children’s involvement in PA and sports, educational programmes providing clear information on the crucial supportive role of parents should be provided [42–44]. However, the current evidence on family setting highlighted in the framework could mainly reflect perceived importance and modifiability perspectives, rather than providing causality.

The socio-cultural environment setting reinforces the importance of the “Encouragement from significant others” and “Having a companion for physical activity”, which received high rates from experts, and are already evaluated for their association with PA behaviours in previous investigations [20]. Moreover, “Social economic status” has been also highly rated by policy makers and stakeholders for its impact. Social economic status always emerges as a relevant factor when investigating healthy-related behaviours. However, lack of clear associations emerged for adolescents, whilst convincing evidence emerged for adults [21]. A controversial picture emerged if children’s behaviours is investigated across several European countries [45]. In contrast, children and adolescents from low socio-economic status are more likely to engage in unhealthy behaviours, not limited to PA behaviours, but also diet habits, smoking, and drug use [46]. Therefore, further research is required to clarify the role of social economic status when adults act as parents, hence influencing children PA behaviours and sedentary behaviours.

The EU-PAD-A framework provides an upgrade of the previous one [23], demonstrating the need to create a refined concept mapping regarding the determinants of PA behaviours. Specifically, the new EU-PAD-A framework was tailored only for adolescents, highlighted potential settings where adolescent PA behaviours could be adopted, implemented the feasibility and impact ratings from policy makers and stakeholders. Moreover, new and specific determinants were identified as those related to wearables, social media, and inclusive school programmes. The EU-PAD-A framework allowed identifying those settings where research and interventions should be implemented. The framework can be translated in practical implications for a greater promotion of a healthier lifestyle among adolescents, like prioritising inclusive school programmes and facility maintenance in national action plans, and collecting policies and interventions at local, regional, and national level.

However, several limitations of this study should be acknowledged. First, adolescents did not directly participate in any phase of the concept mapping process. This choice was intentional, as the framework was designed to synthesize determinants from the perspective of experienced adult stakeholders with expertise in physical activity research, policy and practice. Nevertheless, the absence of adolescent opinions limits the integration of lived experiences and should be addressed in future work. Second, the ratings and sorting tasks relied on expert judgement and therefore reflect a degree of intrinsic subjectivity, which may also be influenced by prevailing European public health and policy agendas. In addition, although experts from both EU and non-EU countries were involved across all phases, participants from EU countries were more strongly represented, reflecting the European scope and structure of the COST Action. While this strengthens the relevance of the EU-PAD-A framework for European contexts, it may reduce its direct generalizability to non-European settings. Third, despite the inclusion of a broad range of professional roles and sectors, academic and research experts constituted a larger proportion of participants, particularly during the brainstorming phase, compared with non-academic stakeholders. This imbalance may have influenced the prioritization of determinants through a stronger research-oriented lens. However, the involvement of governance, education, health, and industry professionals in the sorting and rating phases partially mitigates this risk by incorporating more practice- and policy-oriented perspectives. A further potential source of bias relates to the sample of policy makers and stakeholders, who were recruited through professional networks and voluntary participation and were predominantly drawn from the health and sport sectors. This may have shaped perceptions of impact and feasibility toward determinants aligned with these domains. Finally, subgroup analyses based on parental status were not conducted. As shown in Table 2, participants with and without parental or guardian responsibilities were represented across all GCM phases, although with varying proportions. However, potential differences in perspectives between these groups were not formally examined. Consequently, distinct viewpoints related to lived parenting experience, family dynamics and the support of adolescents’ physical activity behaviours may not have been fully captured.

Future research should therefore aim to: (a) triangulate expert-driven frameworks with adolescent-centred participatory approaches; (b) further expand the involvement of non-academic stakeholders from social, youth and community sectors; and (c) explore subgroup-specific perspectives, including parental roles, to refine and contextualise the EU-PAD-A framework.

## Conclusions

The EU-PAD-A framework provides new insights on the most influential clusters and determinants for adolescents’ behaviours that could be translated in actions and could be further investigated. School, family, and socio-cultural environment emerged as critical settings where the adolescents’ behaviour towards a healthy lifestyle needs to be embedded and developed. The analysis of impact and feasibility from policy makers and stakeholders could guide future research and initiatives across Europe. The emerging settings require the development of specific initiatives centred on a transdisciplinary, multi-settings, and dynamic approach to impact the adolescents’ behaviours towards the increase of PA level and the reduction of sedentary behaviours.

## Supplementary Information

Supplementary Material 1.

Supplementary Material 2.

Supplementary Material 3.

Supplementary Material 4.

Supplementary Material 5.

Supplementary Material 6.

## Data Availability

The datasets used and/or analysed during the current study are available from the corresponding author on reasonable request.

## Abbreviations

PA: Physical Activity
EU-PAD-A: EUropean Physical Activity Determinants framework for Adolescents
DE-PASS: DEterminants of Physical ActivitieS in Settings
COST: European Cooperation in Science and Technology
DEDIPAC: DEterminants of DIet and Physical ACtivity
BESt: Best Evidence Statements
GCM: Group Concept Mapping
SPSS: Statistical Package for the Social Sciences
EU: European Union
WHO: World Health Organization
