# Supplementary material for: The development of the EUropean Physical Activity Determinants framework for Adolescents (EU-PAD-A): a mixed-methods concept mapping study within the DE-PASS COST action
Source: Int J Behav Nutr Phys Act. 2026 Feb 5;23:22. doi: 10.1186/s12966-026-01878-0 (PMC12973781; doi:10.1186/s12966-026-01878-0)
Supplement: Supplementary file 1 — Supplementary Material 1. [file 12966_2026_1878_MOESM1_ESM.docx]

**Table S1**. List of the 110 determinants divided by each cluster and with the average value for importance, modifiability, bridging, and Go-zones quadrant position.

|  | | Mean rating ^a^ | |  |  |
| --- | --- | --- | --- | --- | --- |
| Cluster name and statements | | Importance | Modifiability | Bridging | Go-zones  Quadrant ^b^ |
| **Determinant Number** | **Cluster 1: Psychological and Motivational Traits** | **Avg 4.46** | **Avg 4.05** | **Avg 0.22** |  |
| 1 | Knowledge/awareness of health benefits | 4.18 | 4.85 | 0.30 | 4 |
| 6 | Perception of benefits of sport | 4.62 | 4.61 | 0.31 | 4 |
| 23 | Self efficacy | 4.47 | 4.12 | 0.12 | 4 |
| 24 | Self confidence | 4.70 | 4.45 | 0.13 | 4 |
| 25 | Self esteem | 4.66 | 4.11 | 0.13 | 4 |
| 26 | Self competence | 4.32 | 4.20 | 0.15 | 4 |
| 27 | Self image | 4.40 | 4.12 | 0.12 | 4 |
| 28 | Body image | 4.21 | 4.06 | 0.14 | 4 |
| 31 | Previous experiences in physical activity | 4.42 | 2.52 | 0.36 | 2 |
| 32 | Previous experiences in sport | 4.39 | 2.48 | 0.34 | 2 |
| 62 | Depressive symptoms | 4.64 | 3.90 | 0.13 | 4 |
| 63 | Anxiety symptoms | 4.20 | 4.02 | 0.12 | 4 |
| 65 | Fear of Injuries | 3.36 | 3.98 | 0.14 | 3 |
| 66 | Physical activities perceived benefits | 4.71 | 4.54 | 0.38 | 4 |
| 67 | Personality traits | 4.14 | 2.79 | 0.15 | 2 |
| 86 | Autonomy/independence | 4.20 | 4.00 | 0.19 | 4 |
| 87 | Enjoyment | 5.22 | 4.59 | 0.21 | 4 |
| 88 | Psychological well-being | 4.80 | 4.43 | 0.13 | 4 |
| 89 | Intrinsic motivation | 5.07 | 4.04 | 0.17 | 4 |
| 90 | Attitude | 4.57 | 4.17 | 0.18 | 4 |
| 91 | Belief about physical activity | 4.59 | 4.50 | 0.28 | 4 |
| 93 | Stress | 4.26 | 4.03 | 0.15 | 4 |
| 101 | Extrinsic motivation | 4.01 | 4.17 | 0.39 | 3 |
| 103 | Intention to be physically active | 4.73 | 4.65 | 0.22 | 4 |
| 104 | Perceived barriers to physical activity | 4.38 | 4.03 | 0.53 | 4 |
|  | **Cluster 2: Biological Features and Lifestyle** | **Avg 4.02** | **Avg. 3.06** | **Avg 0.22** |  |
| 3 | Age | 3.23 | 1.34 | 0.21 | 1 |
| 4 | Gender | 2.56 | 1.50 | 0.27 | 1 |
| 5 | Growth/maturation | 4.00 | 2.65 | 0.20 | 1 |
| 8 | Genetic aspects | 3.37 | 1.44 | 0.28 | 1 |
| 10 | Time-management skills | 4.11 | 4.28 | 0.23 | 4 |
| 29 | Overall physical fitness levels | 4.41 | 4.41 | 0.17 | 4 |
| 30 | Fundamental movement skills | 4.49 | 3.90 | 0.21 | 4 |
| 33 | Body weight | 4.19 | 4.00 | 0.19 | 4 |
| 35 | Physical disability | 4.59 | 2.05 | 0.31 | 2 |
| 36 | Mental/intellectual disability | 4.59 | 2.07 | 0.18 | 2 |
| 38 | Health status | 4.95 | 4.05 | 0.17 | 4 |
| 39 | Sleep behaviours and habits | 4.19 | 4.32 | 0.34 | 4 |
| 92 | Pain/fatigue/weakness | 4.28 | 3.71 | 0.19 | 4 |
| 106 | Physical well-being | 4.81 | 4.37 | 0.16 | 4 |
| 109 | Stature | 2.61 | 1.84 | 0.21 | 1 |
|  | **Cluster 3: Family Influence** | **Avg. 4.11** | **Avg. 3.42** | **Avg 0.19** |  |
| 2 | Family knowledge of physical activity | 4.24 | 4.06 | 0.16 | 4 |
| 19 | Communication and interactions within the family | 4.13 | 4.21 | 0.16 | 4 |
| 21 | Educational level of the family | 4.17 | 2.55 | 0.22 | 2 |
| 45 | Physical activity engagement of the family | 4.46 | 3.75 | 0.17 | 4 |
| 49 | Parental support | 4.89 | 4.07 | 0.19 | 4 |
| 64 | Adolescent parenthood | 3.98 | 2.81 | 0.24 | 1 |
| 98 | Occupation of the parents | 3.52 | 2.25 | 0.19 | 1 |
| 100 | Family's norms and rules | 4.48 | 3.54 | 0.17 | 2 |
| 105 | Participating in physical activity together with the family | 4.19 | 4.07 | 0.17 | 4 |
| 107 | Parents' body weight | 3.05 | 2.87 | 0.21 | 1 |
|  | **Cluster 4: Socio-Cultural and Media Context** | **Avg. 3.37** | **Avg. 3.27** | **Avg 0.67** |  |
| 7 | Ethnicity | 2.56 | 1.42 | 0.56 | 1 |
| 20 | Cultural factors | 3.94 | 2.71 | 0.61 | 1 |
| 22 | Social economic status | 4.27 | 2.56 | 0.62 | 2 |
| 69 | Exposure to social media | 3.65 | 4.17 | 0.66 | 3 |
| 82 | Language difficulties | 2.92 | 3.65 | 0.58 | 3 |
| 85 | Mass media campaigns and advertisements | 3.83 | 4.34 | 0.81 | 3 |
| 102 | Use of digital devices | 3.51 | 4.27 | 0.64 | 3 |
| 108 | Usage of fitness tracker devices | 3.19 | 4.35 | 1.00 | 3 |
| 110 | Religion | 2.42 | 1.99 | 0.59 | 1 |
|  | **Cluster 5: Dietary and Substance Use** | **Avg. 3.96** | **Avg 4.47** | **Avg 0.43** |  |
| 34 | Dietary behaviours and habits | 4.14 | 4.55 | 0.42 | 4 |
| 37 | Recreational drugs use | 4.03 | 4.37 | 0.42 | 3 |
| 40 | Smoking behaviours and habits | 3.87 | 4.40 | 0.46 | 3 |
| 70 | Alcohol consumption | 3.83 | 4.58 | 0.42 | 3 |
|  | **Cluster 6: Social Supporting** | **Avg 4.47** | **Avg 4.03** | **Avg 0.58** |  |
| 48 | Support from peers | 4.39 | 3.97 | 0.54 | 4 |
| 68 | Bullying | 4.21 | 4.08 | 0.66 | 4 |
| 94 | Encouragement from significant others | 4.67 | 4.10 | 0.50 | 4 |
| 95 | Having a companion for physical activity | 4.57 | 4.30 | 0.59 | 4 |
| 96 | Social influences | 4.54 | 3.92 | 0.56 | 4 |
| 97 | Social inclusion | 4.53 | 4.22 | 0.58 | 4 |
| 99 | Role models | 4.38 | 3.65 | 0.61 | 4 |
|  | **Cluster 7: Policy Domain** | **Avg. 3.97** | **Avg. 3.54** | **Avg 0.51** |  |
| 9 | Incentives to be physically active | 4.30 | 4.68 | 0.64 | 4 |
| 41 | Physical activity policies | 4.49 | 4.09 | 0.58 | 4 |
| 42 | Sporting events accessibility | 4.04 | 3.85 | 0.35 | 3 |
| 47 | Governing bodies support | 4.13 | 3.80 | 0.54 | 4 |
| 50 | Distance from home to school | 3.49 | 2.30 | 0.33 | 1 |
| 54 | Gym memberships cost | 3.91 | 3.39 | 0.32 | 1 |
| 61 | Presence of physical activity equipment and facilities at home | 3.89 | 3.78 | 0.69 | 3 |
| 71 | Size and availability of backyard space at home | 3.50 | 2.41 | 0.62 | 1 |
|  | **Cluster 8: Physical Environment and Accessibility** | **Avg. 3.82** | **Avg. 3.16** | **Avg 0.10** |  |
| 51 | Proximity of parks | 4.04 | 2.68 | 0.02 | 1 |
| 55 | Weather conditions | 3.51 | 1.67 | 0.09 | 1 |
| 56 | Air pollution | 3.38 | 2.79 | 0.22 | 1 |
| 57 | Noise in the environment | 2.71 | 3.02 | 0.11 | 1 |
| 58 | Availability of indoor and outdoor physical activity facilities | 4.87 | 3.93 | 0.10 | 4 |
| 59 | Indoor and outdoor sports facilities availability | 4.76 | 3.73 | 0.14 | 4 |
| 74 | Active means of transportation use | 4.02 | 3.67 | 0.23 | 3 |
| 75 | Environmental barriers to active travel | 3.91 | 3.59 | 0.07 | 1 |
| 76 | Streets characteristics | 3.56 | 3.14 | 0.02 | 1 |
| 77 | Walkability and safety of sidewalks/trails | 3.88 | 3.87 | 0.00 | 3 |
| 78 | Access to public transport system | 3.89 | 3.53 | 0.10 | 1 |
| 79 | Neighbourhood safety | 4.08 | 3.45 | 0.12 | 2 |
| 80 | Population/residential density | 3.08 | 1.98 | 0.11 | 1 |
| 84 | Urban design and land use | 3.82 | 3.26 | 0.01 | 1 |
|  | **Cluster 9: School and Educational Environment** | **Avg. 4.05** | **Avg. 3.90** | **Avg 0.24** |  |
| 11 | Inclusive programs in school | 4.56 | 4.43 | 0.17 | 4 |
| 12 | School culture | 4.37 | 3.76 | 0.17 | 4 |
| 13 | Proportion of time spent in physical education in comparison to other school subjects | 4.16 | 3.96 | 0.06 | 4 |
| 14 | Active classroom breaks | 3.95 | 4.25 | 0.05 | 3 |
| 15 | Amount of school recess time | 3.57 | 3.70 | 0.05 | 3 |
| 16 | Provision of sports clubs in schools | 4.24 | 4.17 | 0.24 | 4 |
| 17 | Teachers' beliefs about physical activity | 4.21 | 4.14 | 0.12 | 4 |
| 18 | Teachers' beliefs about sport | 4.17 | 4.02 | 0.13 | 4 |
| 43 | Teachers' guidance | 4.14 | 4.07 | 0.17 | 4 |
| 44 | Coaches' assistance | 4.31 | 4.33 | 0.51 | 4 |
| 46 | Higher level education and research centres' support | 3.88 | 3.68 | 0.53 | 3 |
| 52 | Cost of extracurricular physical activities in school | 4.21 | 3.85 | 0.25 | 4 |
| 53 | Expenses related to extracurricular sport in school | 4.29 | 3.80 | 0.25 | 4 |
| 60 | Maintenance of school facilities and equipment | 4.28 | 4.08 | 0.35 | 4 |
| 72 | Availability of family transportation | 3.89 | 3.25 | 0.65 | 1 |
| 73 | Provision of school facilities resources | 4.31 | 3.87 | 0.26 | 4 |
| 81 | Homework | 3.57 | 3.81 | 0.30 | 3 |
| 83 | Class size | 2.88 | 3.09 | 0.10 | 1 |
