# Supplementary material for: The development of the EUropean Physical Activity Determinants framework for Adolescents (EU-PAD-A): a mixed-methods concept mapping study within the DE-PASS COST action
Source: Int J Behav Nutr Phys Act. 2026 Feb 5;23:22. doi: 10.1186/s12966-026-01878-0 (PMC12973781; doi:10.1186/s12966-026-01878-0)
Supplement: Supplementary file 2 — Supplementary Material 2. [file 12966_2026_1878_MOESM2_ESM.docx]

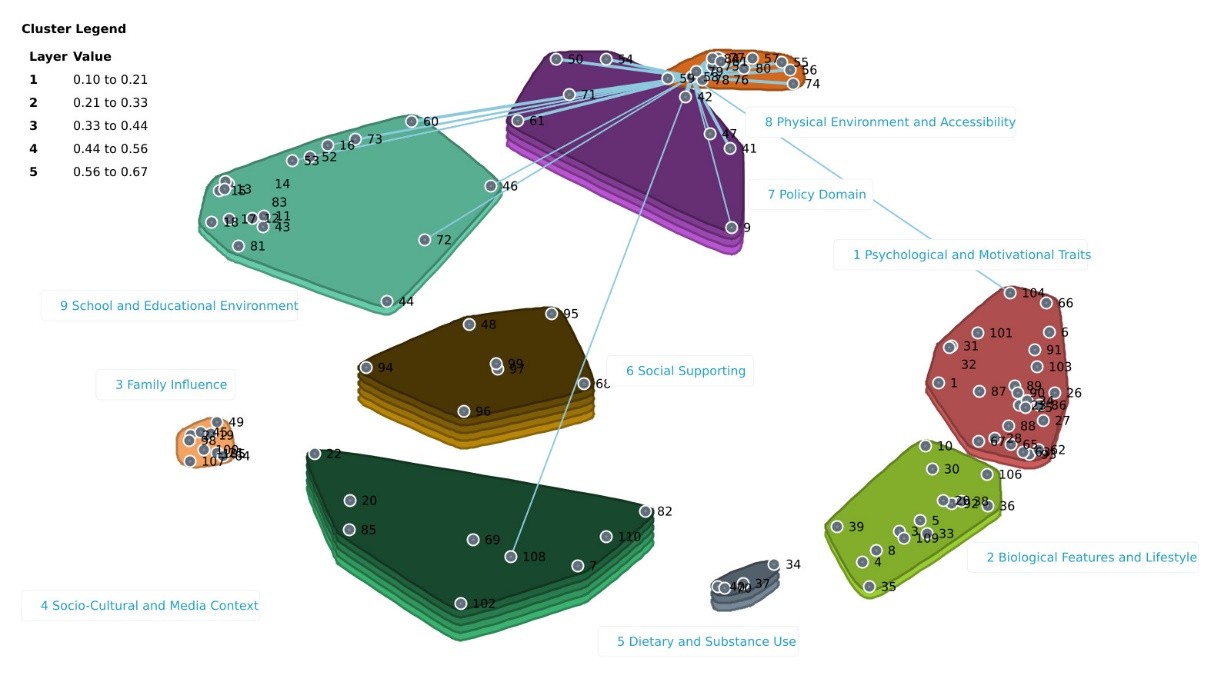


**Figure S3**. Spanning analysis of “Availability of indoor and outdoor physical activity facilities” as an anchor (bridge value 0.10) was sorted 74 times with “Indoor and outdoor sports facilities” within the same cluster and 55 times with “Sporting events accessibility” from Cluster 7.
