# Supplementary material for: The development of the EUropean Physical Activity Determinants framework for Adolescents (EU-PAD-A): a mixed-methods concept mapping study within the DE-PASS COST action
Source: Int J Behav Nutr Phys Act. 2026 Feb 5;23:22. doi: 10.1186/s12966-026-01878-0 (PMC12973781; doi:10.1186/s12966-026-01878-0)
Supplement: Supplementary file 3 — Supplementary Material 3. [file 12966_2026_1878_MOESM3_ESM.docx]

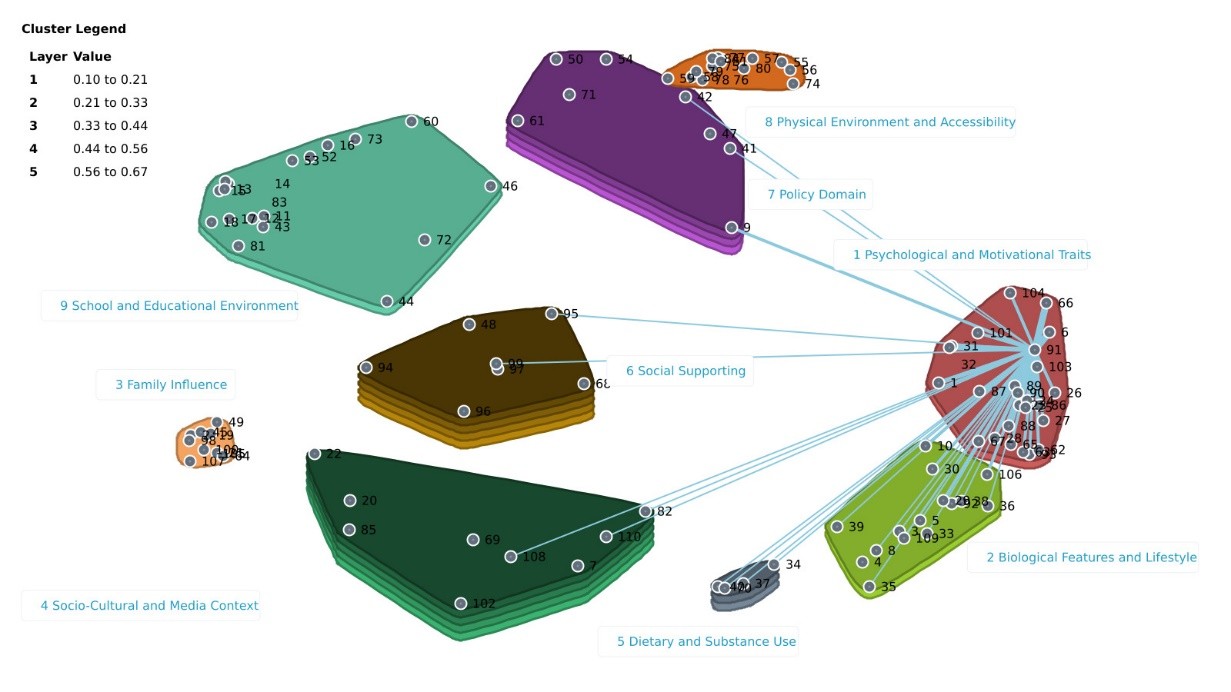


**Figure S4**. Spanning analysis of “Belief about physical activity”, as an anchor (bridge value 0.28) was sorted 60 times with “Physical activities perceived benefits” and “Perception of benefits of sport” within the same cluster and 34 times with “Physical well-being” from Cluster 2.
