# Supplementary material for: The development of the EUropean Physical Activity Determinants framework for Adolescents (EU-PAD-A): a mixed-methods concept mapping study within the DE-PASS COST action
Source: Int J Behav Nutr Phys Act. 2026 Feb 5;23:22. doi: 10.1186/s12966-026-01878-0 (PMC12973781; doi:10.1186/s12966-026-01878-0)
Supplement: Supplementary file 4 — Supplementary Material 4. [file 12966_2026_1878_MOESM4_ESM.docx]

**
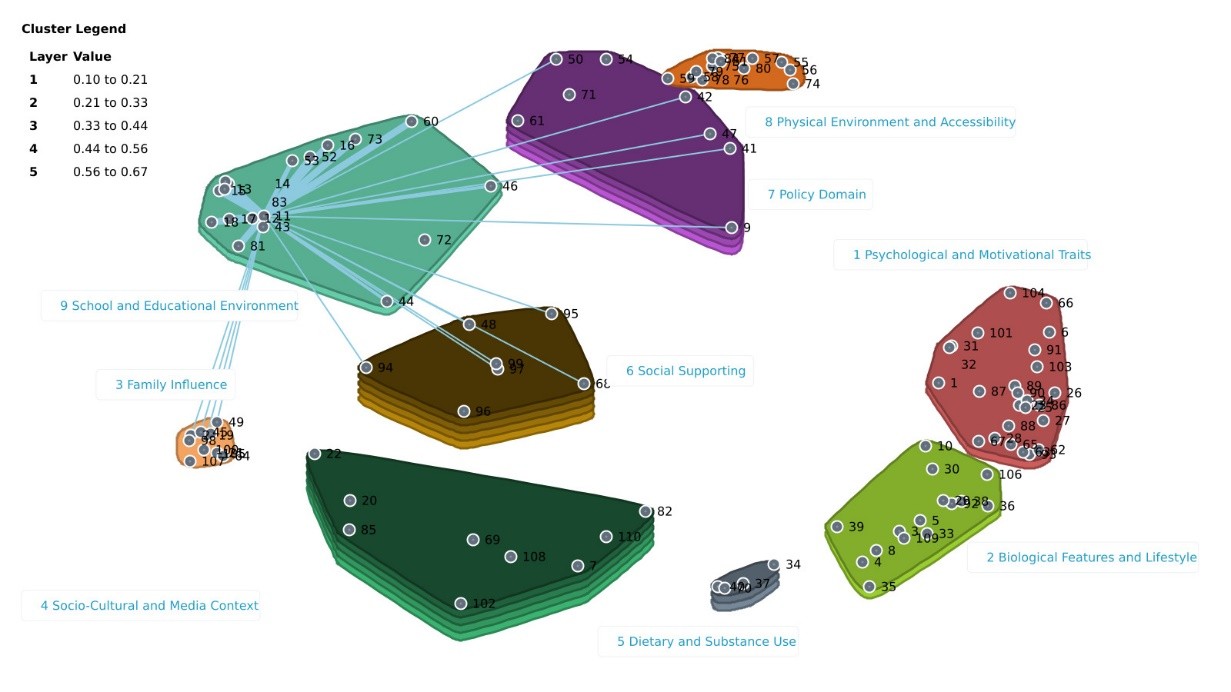
**

**Figure S5**. Spanning analysis of “Inclusive programs in schools” as an anchor (bridge value 0.17) was sorted 66 times with “Provision of sports clubs in schools” within the same cluster and 23 times with “Distance from home to school” from Cluster 7.
