# Supplementary material for: The development of the EUropean Physical Activity Determinants framework for Adolescents (EU-PAD-A): a mixed-methods concept mapping study within the DE-PASS COST action
Source: Int J Behav Nutr Phys Act. 2026 Feb 5;23:22. doi: 10.1186/s12966-026-01878-0 (PMC12973781; doi:10.1186/s12966-026-01878-0)
Supplement: Supplementary file 5 — Supplementary Material 5. [file 12966_2026_1878_MOESM5_ESM.docx]

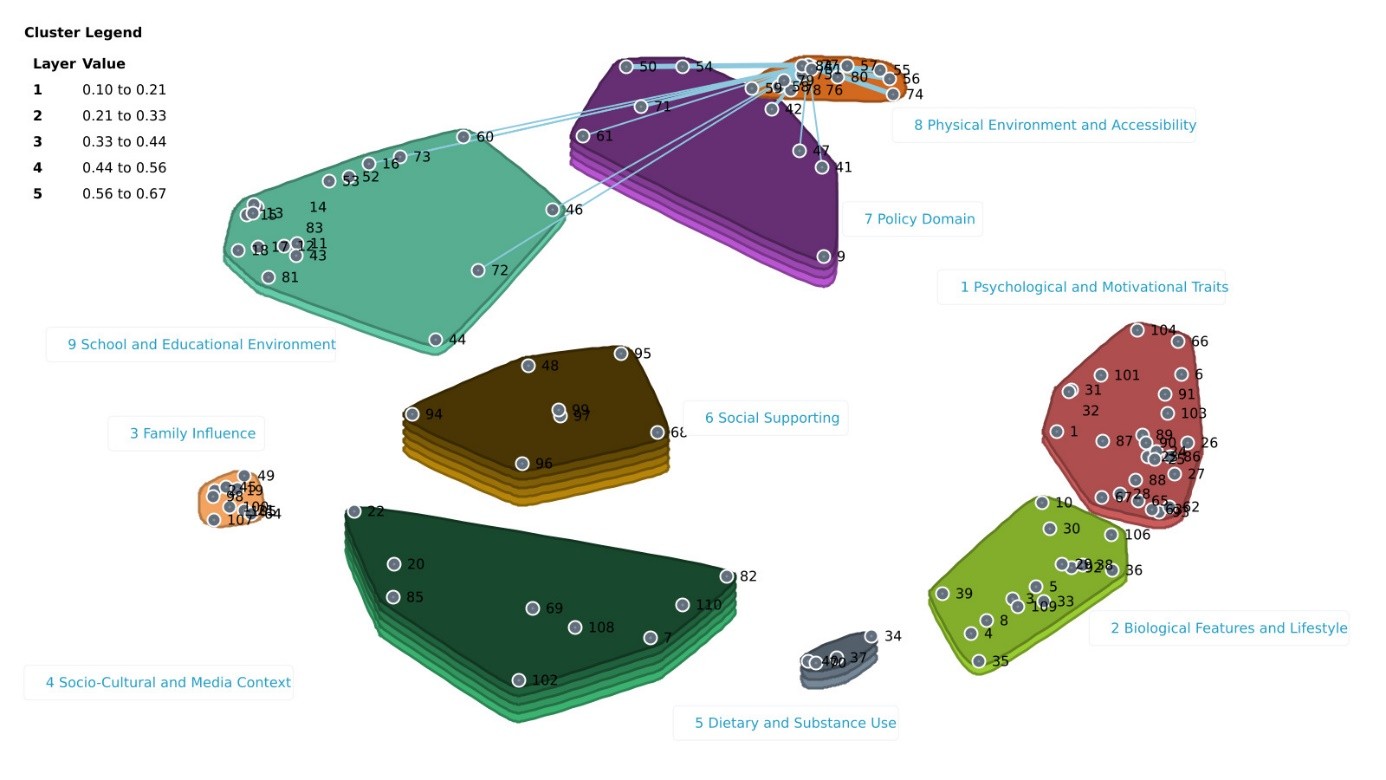


**Figure S2**. Spanning analysis of Cluster 8 “Physical Environment and Accessibility”. Example of an Anchor determinant “Walkability and safety of sidewalks/trails”.
